# Editorial Note: Prevalence of Chinook salmon is higher for southern than for northern resident killer whales in summer hot-spot feeding areas

**DOI:** 10.1371/journal.pone.0353511

**Published:** 2026-07-13

**Authors:** The PLOS One Editors

After publication of this article [1], concerns were raised by a reader about the interpretation of the study’s findings and the information provided regarding killer whale prey availability and foraging activity. After editorial follow-up with the authors and consulting a member of the Editorial Board with expertise in population data analysis, the *PLOS One* Editors assessed these concerns and consider that the article’s findings remain supported. This Editorial Note is issued to clarify points raised during post‑publication assessment and to provide additional information regarding comparisons with earlier research, study limitations, and the rationale for site selection.

## 1. Comparison with previous research

Based on input received during editorial follow-up, the *PLOS One* Editors consider that the study reported in [1] is complementary to the body of research suggesting food limitation in southern resident killer whales (SRKWs) [2–4]. The member of the Editorial Board noted that divergence between the study reported in [1] and expectations based on this prior research highlights the need for continued investigation into the seasonal and regional context of prey availability for SRKWs.

The last author stated that they are in agreement with prior findings that SRKWs are food limited, but noted that the study in [1] was designed to determine whether the food shortage was occurring during the summer when SRKWs typically travel north to the Salish Sea to feed, or whether it could be occurring at other limiting times of year such as winter or spring. They further noted that they consider the findings reported in [1] augment previous knowledge, but direct comparisons between studies are limited owing to differing study scopes, data types, and seasonal coverage.

## 2. Study limitations

The study relies in part on information obtained from whale‑watching professionals and professional sport fishermen for site selection; a member of the Editorial Board noted this may introduce potential bias affecting the representativeness of selected foraging hot‑spot locations. It was further noted that some of the feeding hotspot information used in this study [1] differed compared to prior reports identifying feeding and foraging areas [5–7]. The member of the Editorial Board noted that further research will be required to validate information obtained from whale-watching professionals and fishermen. Other key limitations related to undersampling foraging hot spots relative to the full spatial and temporal ranges used by resident killer whales, and constraints associated with conducting a single-season survey are discussed in [1].

Based on information provided by the last author, this notice provides the following additional information regarding study site selection:

Two study sites were selected, the Salish Sea and North Island Waters, because they are important and well-documented feeding areas used by resident killer whales during summer [8–10] that have historically been the focus of the majority of field studies on both SRKWs and NRKWs [11–16]. Hot-spot feeding locations within each region were identified through consultations with experienced whale-watching professionals and full-time sport fishermen. These individuals were asked to annotate independent nautical charts to respectively indicate areas of high killer whale feeding activity or areas of high Chinook salmon numbers. Congruence between the independently created maps provided validation of site selection. The authors consider their consultations with fishermen and whale‑watching professionals a major strength that informed the study’s design and findings.
